# The mitochondrial genome of a leaf insect *Phyllium westwoodii* (Phasmatodea: Phylliidae) in Southeast Asia

**DOI:** 10.1080/23802359.2021.1886014

**Published:** 2021-03-15

**Authors:** Zhiwei Dong, Jun Li, Jinwu He, Guichun Liu, Chuyang Mao, Ruoping Zhao, Xueyan Li

**Affiliations:** a State Key Laboratory of Genetic Resources and Evolution, Kunming Institute of Zoology, Chinese Academy of Sciences, Kunming, China; b School of Ecology and Environment, Northwestern Polytechnical University, Xi’an, China

**Keywords:** Mitogenome, leaf insect, *Phyllium westwoodii*, phylogenetic analysis

## Abstract

The nearly complete mitochondrial genome (mitogenome) of *Phyllium westwoodii*, a typical leaf mimic insect in Phasmatodea, was obtained in this study. This mitogenome is 17,222 bp in length and contains 13 protein-coding genes (PCGs), 22 transfer RNA genes (tRNAs), two ribosomal RNA genes (rRNAs) and almost complete control regions. All PCGs initiate with ‘ATN’ except for *NAD4L* that uses ‘TTG’ as the start codon, and terminate with ‘TAA’ except for *COX2* that uses a single ‘T’ residue as the stop codon. The phylogenetic analysis based on the concatenated sequences of 13 PCGs and two rRNAs shows that *P*. *westwoodii* is closer to *Phyllium tibetense* than *Phyllium giganteum*.

The Phasmatodea is known as stick and leaf insects, and currently contains three suborders, 13 families and more than 3,000 species (Zhang et al. 2011; Bradler et al. 2014). They occur across the tropics and only a few inhabit in temperate areas (Bradler et al. 2014). As typical representatives of Phasmatodea, only a small percentage of extant phasmids (78 extant species belonging to the leaf insects in the Phylliidae) exhibits an extreme form of morphological and behavioral leaf mimicry (Wedmann et al. 2007; Brock et al. 2020), which originated at least 47 million years (Wedmann et al. 2007) . Thus, leaf insects are one of ideal models to investigate the adaptive evolution of leaf mimic traits. However, its genetic basis remains completely unknown. To date, the only available molecular data resources for leaf insects are two mitogenomes deposited in GenBank (Komoto et al. 2011; Zhou et al. 2017).

In the present study, as the first step to dissect *de novo* reference genome of one representative leaf insect, we used Illumina next-generation sequencing (NGS) data to generate the nearly complete mitogenome (with the exception portions of the control region) of *Phyllium westwoodii* (Wood-Mason 1875). This species is one member in Phylliidae and mainly distribute in countries of Southeast Asia, including India (South Andaman Island), Myanmar, China (Yunnan), Thailand, Laos, Vietnam, Sumatra, and Singapore etc. (Hennemann et al. 2009). Male and female adults were collected from Muang Fuang, Nang Ha, Laos (**102°7′3″E; 18°39′12″N**) during June 2017 by Zhiwei Dong and local villagers, and bred in the greenhouse with host plant *Rubus sp*. The voucher specimens (male: KIZ0127554, female: KIZ0127555) are stored in Kunming Natural History Museum of Zoology, Chinese Academy of Sciences. Their offspring (1st instar larvae) was used to extract genomic DNA (gDNA) using a Gentra Puregene Blood kit (Qiagen, Hilden, Germany) based on instructions. Paired-end library (350-bp insert size) was prepared using NEB Next® Ultra DNA Library Prep Kit and sequenced on Illumina HisSeq4000 (Novogene, Beijing, China). 288 Gb total number of bases (SRA number: SRR13336961-SRR13336964) were obtained. After low quality reads were filtered, clean mito-reads were extracted based on homology comparison as previously described (Tang et al. 2014). Mitogenome was assembled using SOAPdenovo-Trans version 1.03 (kmer = 37) (Xie et al. 2014) and subsequently the gaps was filled using GapCloser version 1.12 (Luo et al. 2012). Gene annotation was performed by MITOS2 webserver (http://mitos2.bioinf.uni-leipzig.de/index.py) (Bernt et al. 2013) with manual corrections on cyclization of mitogenome sequence, confirmation of relative position of 37 genes, and the determination of the start and stop codes of 13 protein-coding genes with reference to the mitogenomes (listed as in Figure 1) of Phasmatodea deposited in GenBank.

**Figure 1.**
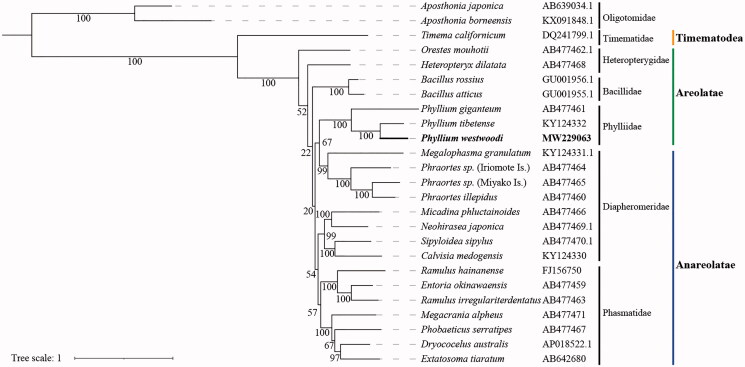
Inferred phylogenetic relationships among Phasmatodea based on the nucleotide sequence of concatenated 13 protein-coding genes (PCGs) and two ribosomal RNA genes (rRNAs) (*rrnL* and *rrnS*) using maximum likelihood (ML) analysis. Both *Aposthonia japonica* and *Aposthonia borneensis* were used as outgroups. The species *Phyllium westwoodii* in this study is highlighted in bold.

The nearly complete mitogenome of *P. westwoodii* totalizes 17,222 bp, which contain 37 typical mitochondrial genes (13 protein-coding genes (PCGs), 22 transfer RNA genes (tRNAs) and two ribosomal RNA genes (rRNAs)) and two unconnected fragments of control region (GenBank accession No. MW229063.1). The total length of all 13 PCGs is 11,131 bp with a strong bias toward A + T (76.35%), and count 64.63% of the whole mitogenome. All PCGs initiate with ‘ATN’ (N represents A, T, G, C) except for *NAD4L* that uses ‘TTG’ as the start codon. Unlike the custom of terminating with ‘TAA’, *COX2* stops with a single ‘T’ as previously reported (Wolstenholme 1992). The 22 tRNA genes range from 62 bp to 71 bp in length. 21 of them have a typical clover-leaf structure except the *trnS1*(gct), in which the dihydrouridine (DHU) arm is replaced by a simple loop that is ubiquitous in most insects (Jiang et al. 2016). The size of large rRNA (*rrnL*) and small rRNA (*rrnS*) is 1,276 bp and 793 bp, respectively.

The mitogenomic sequences (including the nucleotide sequences of 13 PCGs and two rRNAs) of newly generated in this study (*P. westwoodii*), and previously reported from 22 Phasmatodea species (Mikheyev et al. 2017; Zhou et al. 2017) were used to reconstruct phylogenetic tree with *Aposthonia japonica* and *Aposthonia borneensis* (Embioptera: Oligotomidae) as outgroups. 13 PCGs and two rRNA were aligned by MEGA-X (Kumar et al. 2018), respectively. TrimAl v1.4.rev22 (Capella-Gutierrez et al. 2009) was used to remove unreliably aligned sites (gt = 0.5). The sequence matrix with 13,171 aligned nucleotide sites were concatenated using SequenceMatrix v1. 8 (Vaidya et al. 2011) prior to phylogenetic analyses. PartitionFinder v2.1.1 (Lanfear et al. 2017) was run twice to select best-fit partitioning schemes and models of evolution for nucleotide (GTR + G + I). Maximum likelihood (ML) tree was reconstructed using RAxML v8.2.10 (Stamatakis 2014) based on the rapid bootstrap (BS) algorithm with 1000 bootstrap replicates. Our phylogenetic analysis shows that *P. westwoodii* is closer to *Phyllium tibetense* than *Phyllium giganteum* (Figure 1).

## Data Availability

The genome sequence data that support the findings of this study are openly available in GenBank of NCBI at (https://www.ncbi.nlm.nih.gov) under the accession no. MW229063.1. The associated BioProject, Bio-Sample, and SRA numbers are PRJNA682332, SAMN16988089 and SRR13336961-SRR13336964, respectively.
